# Supplementary material for: Experimental and analytical pipeline for sub-genomic RNA landscape of coronavirus by Nanopore sequencer
Source: Microbiol Spectr. 2024 Mar 14;12(4):e03954-23. doi: 10.1128/spectrum.03954-23 (PMC10986531; doi:10.1128/spectrum.03954-23)
Supplement: Fig. S1-S8, Tables S1 and S2 — Supplemental figures and tables. [file spectrum.03954-23-s0001.pdf]

Experimental and analytical pipeline for sub-genomic RNA landscape of coronavirus by Nanopore sequencer

Bo-Jia Chen[1], Ching-Hung Lin[2], Hung-Yi Wu[2], James J. Cai[3], Day-Yu Chao[1,4,5]

[1] Doctoral Program in Microbial Genomics, National Chung Hsing University and Academia Sinica, Taichung 402, Taiwan

[2]Graduate Institute of Veterinary Pathobiology, College of Veterinary Medicine, National Chung Hsing University, Taichung 40227, Taiwan;

[3]Department of Veterinary Integrative Biosciences, Texas A&M University, College Station, TX 77843, USA

[4]Graduate Institute of Microbiology and Public Health, College of Veterinary Medicine, National Chung Hsing University, Taichung City 402, Taiwan

[5]Department of Post-Baccalaureate Medicine, College of Medicine, National Chung Hsing University, Taichung City 402, Taiwan

Supplementary Materials

8 figures and 2 tables

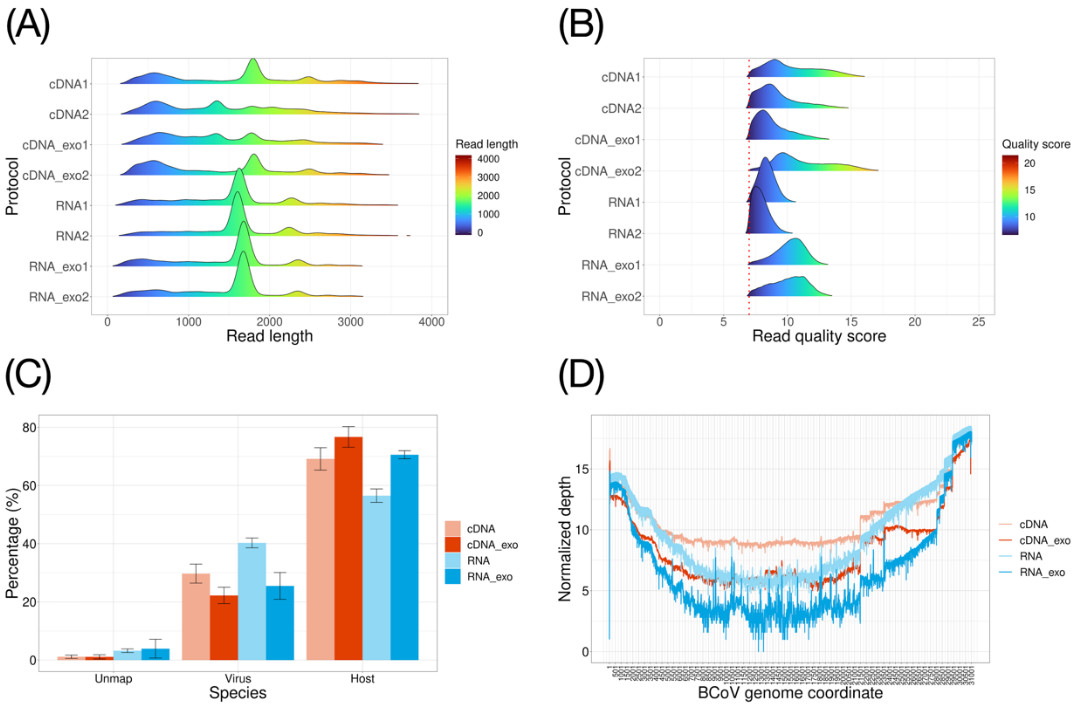

Supplementary Figure S1. The analysis of the reads generated from four different library construction protocols. (A) The BCoV read length distribution among four protocols with two replicates. (B) The BCoV reads quality score distribution among four protocols with two replicates. (C) The proportion of reads aligned to different hosts among four protocols. ”Virus” indicate bovine coronavirus (BCoV) genome (U00735.2), “Host” indicated Homo sapiens (GRCh38) while “unmapped” indicated the reads assigned to neither of the two references. (D) The BCoV genome coverage and depth of all mapped reads among four protocols.

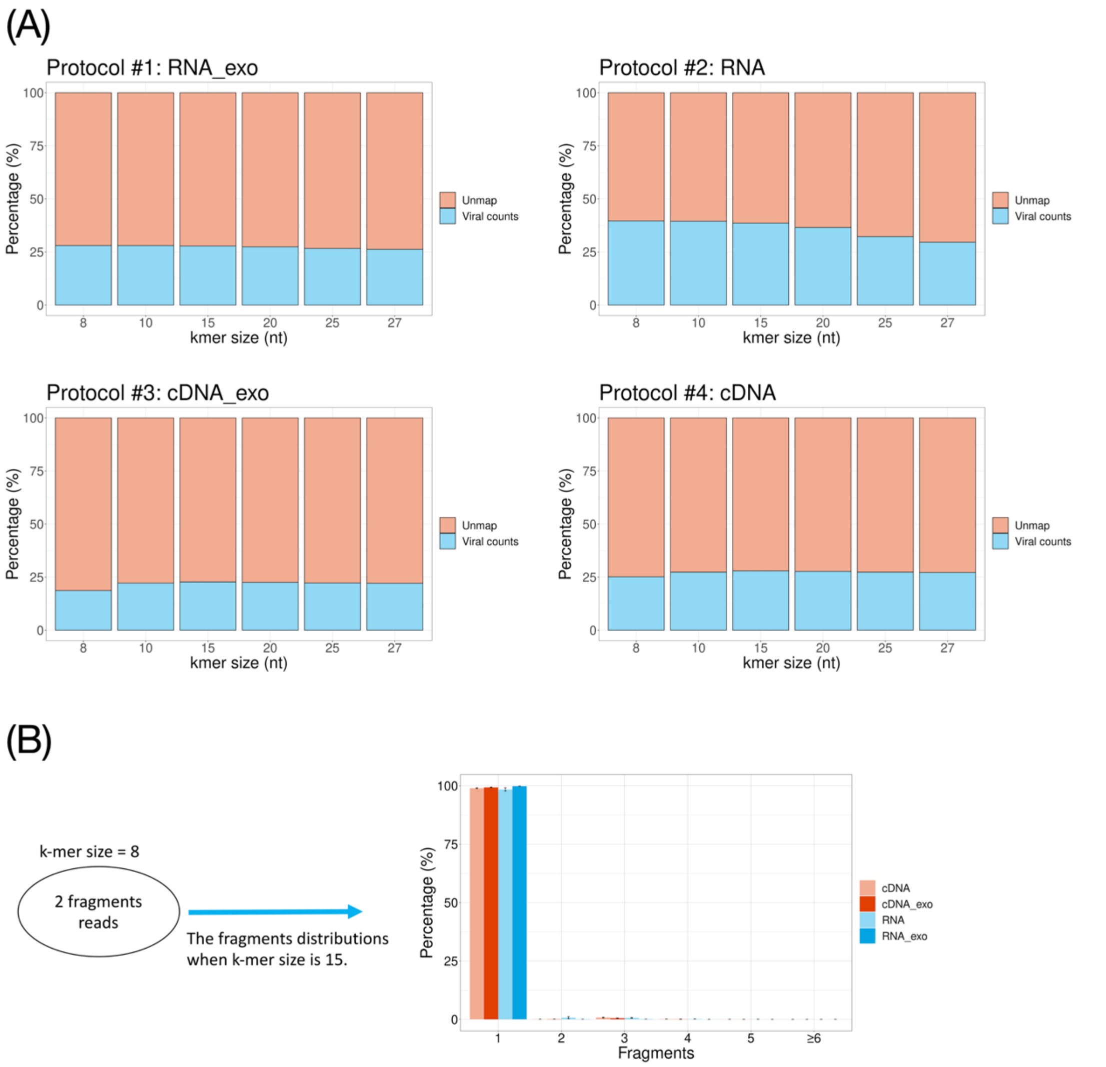

Supplementary Figure S2. (A) Comparison of the proportion of the reads aligned to bovine coronavirus (BCoV) under different k-mer size cut-offs among four different protocols. (B) The percentage of the fragment reassignment using 15-mers as the cutoff among those BCoV-mapped two-fragment reads identified by 8-mers among four protocols. The filtered two-fragments reads mapped to BCoV under k-mer size at 8 were re-aligned under k-mer size cut-off at 15. Nearly 100% were re-assigned as one fragment reads under k-mer size at 15.

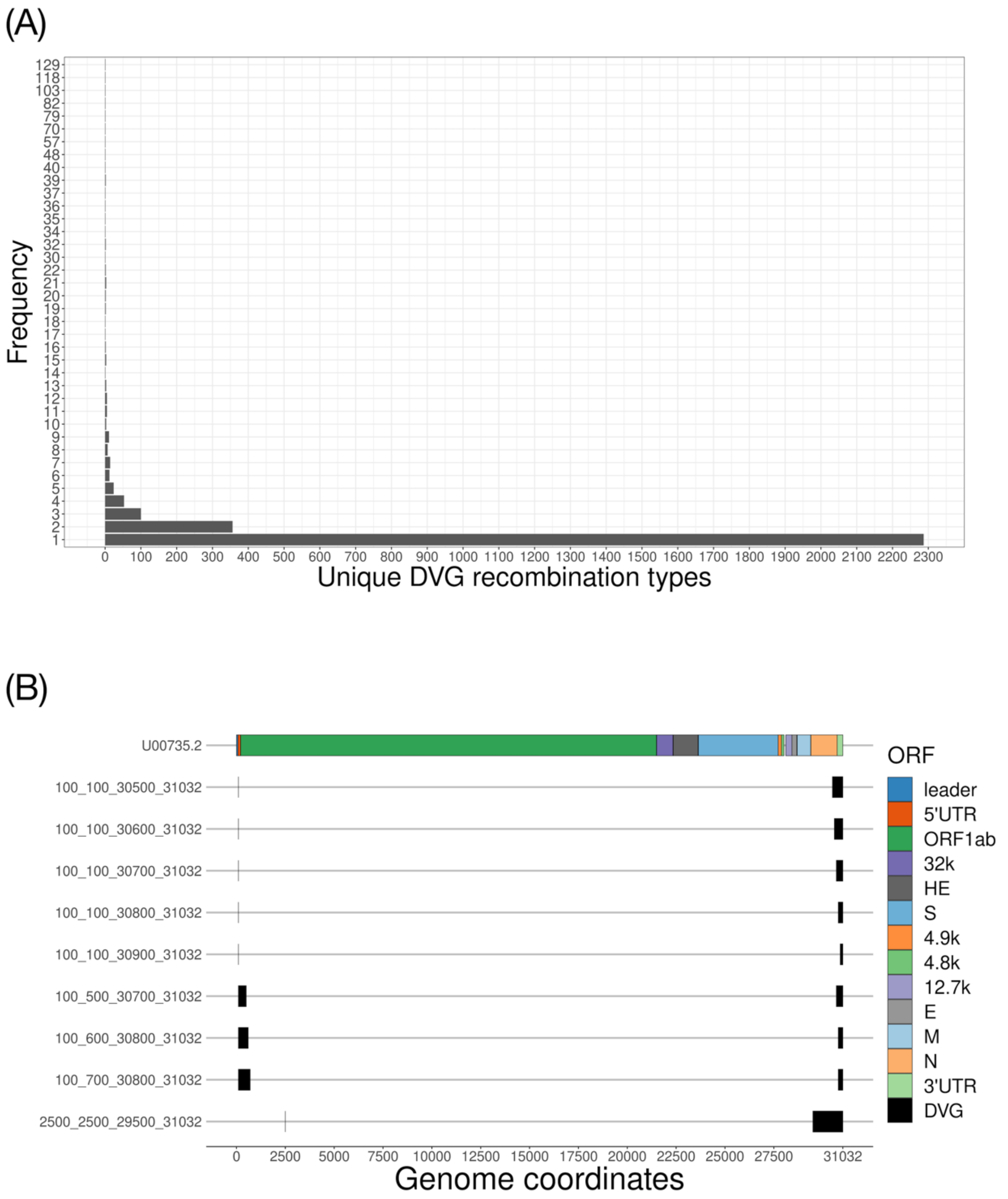

Supplementary Figure S3. The reads abundance (A) and mapped genome locations (B) of DVG types found when the k-mer size was set at 8 but “lost” when the k-mer size was set at 15. “lost” here means the two-fragment reads were re-assigned as one-fragment reads due to different k-mer cutoff.

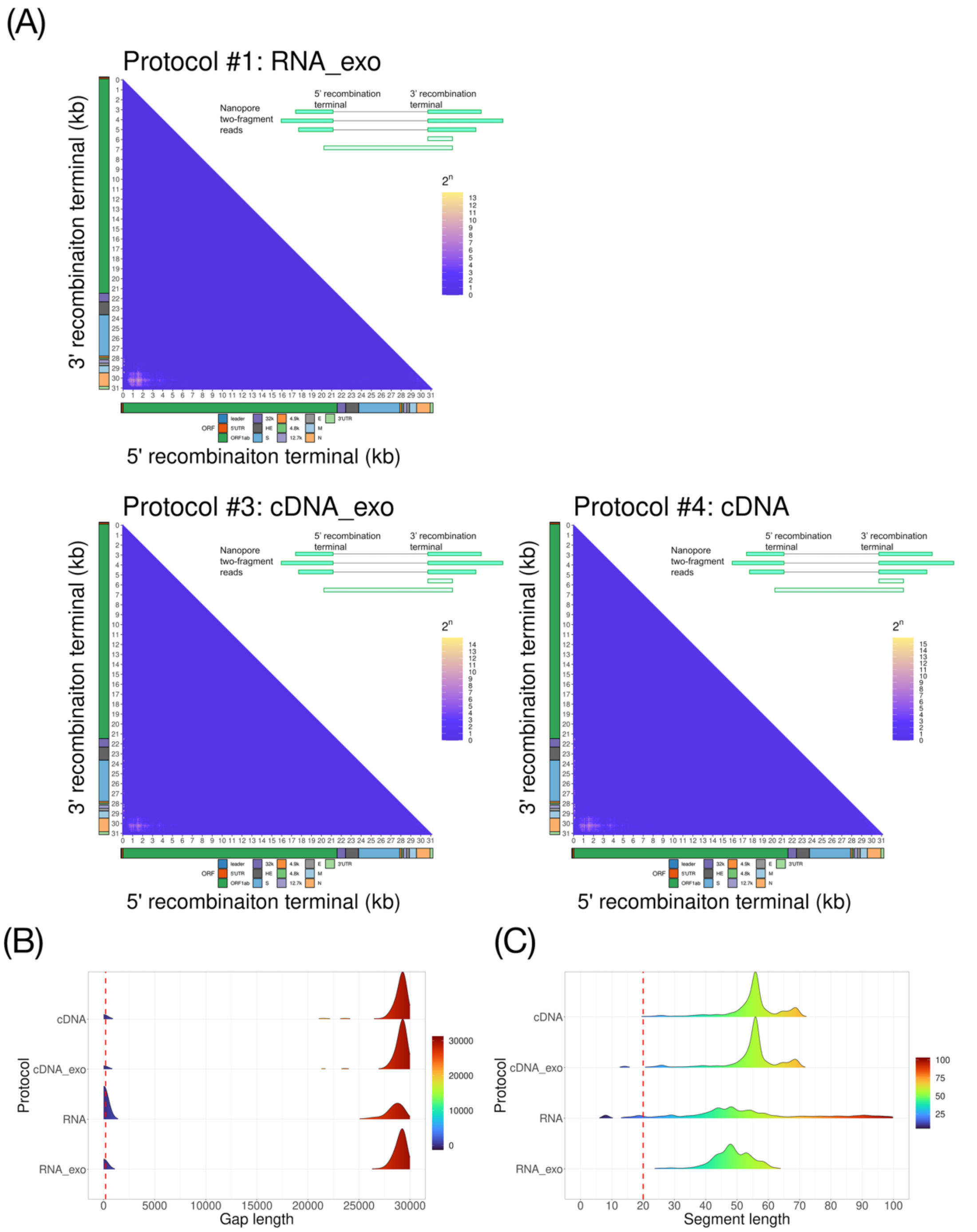

Supplementary Figure S4. (A) The heat map of the recombination sites of two-fragment reads based on Protocol #1, #3 and #4. The up-right corner is the scheme to illustrate the junction terminal of discontinuous viral RNA transcripts. To better visualize, the coordinate of bovine coronavirus (BCoV) genome is binned by 100 base pairs and plot the x and y-axis by kilobase (kb). (B-C) Ridge plot of gap length (B), and segment length (C). The height denotes the relative frequency in each protocol and the color denotes the size of the length displayed by the legend. The segment size is focused on the range of 0 to 100 bps only.

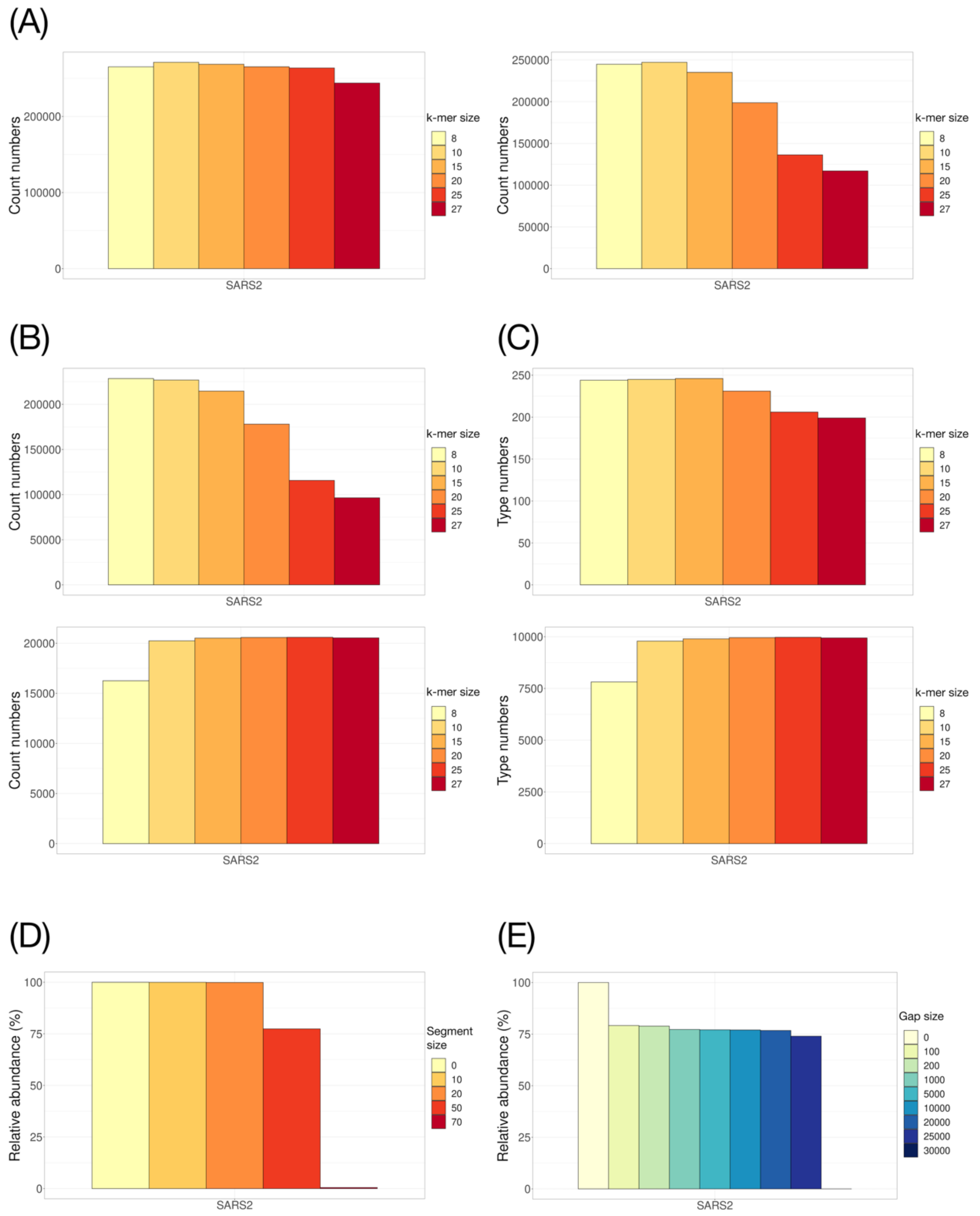

Supplementary Figure S5. The analysis of public DRS SARS2 data (https://doi.org/10.1016/j.cell.2020.04.011) to validate the effect of k-mer size, gap size, and fragment length cutoffs on the discontinuous RNA fragments abundancy. (A) The abundancy distribution of the aligned total RNA reads (left) and 2-fragment reads (right) when using different k-mer size cutoffs for BCoV genome alignment. The numbers of total reads counts (B), and recombination types (C) of the 2-fragment RNA were classified as sgmRNA (B and C upper panel) and DVGs (B and C lower panel). (D) The distribution of the abundance of 2-fragment reads by choosing

different segment sizes (D) and gap sizes (E). The (D) and (E) are transformed into proportion (%) by calculating the counts of 2-fragment reads divided by the filtered reads to total SARS-CoV-2 reads.

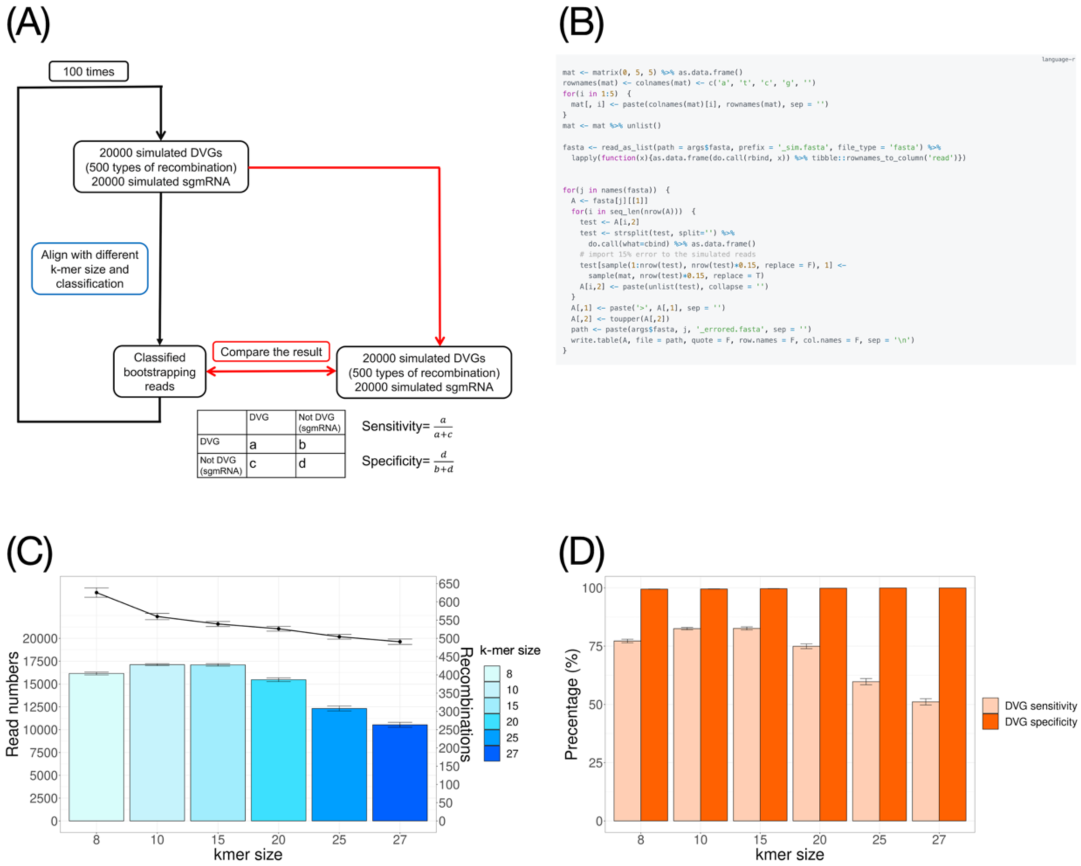

Supplementary Figure S6. Evaluation of the sensitivity and specificity of the proposed bioinformatic pipeline in figure 4 using the simulation data by varying the k-mer size. (A) The graph shows the workflow from how to generate the simulated reads of DVG and sgmRNA libraries to how to examine the sensitivity and specificity of DVGs. (B) The R code shows how to implement a 15 % random error into the simulation reads. (C) The number of reads counts (bar) and recombination types (line) of DVGs after running the bioinformatic pipeline using the simulated reads. (D) The sensitivity and specificity of the proposed bioinformatic pipeline under different k-mer size cutoffs.

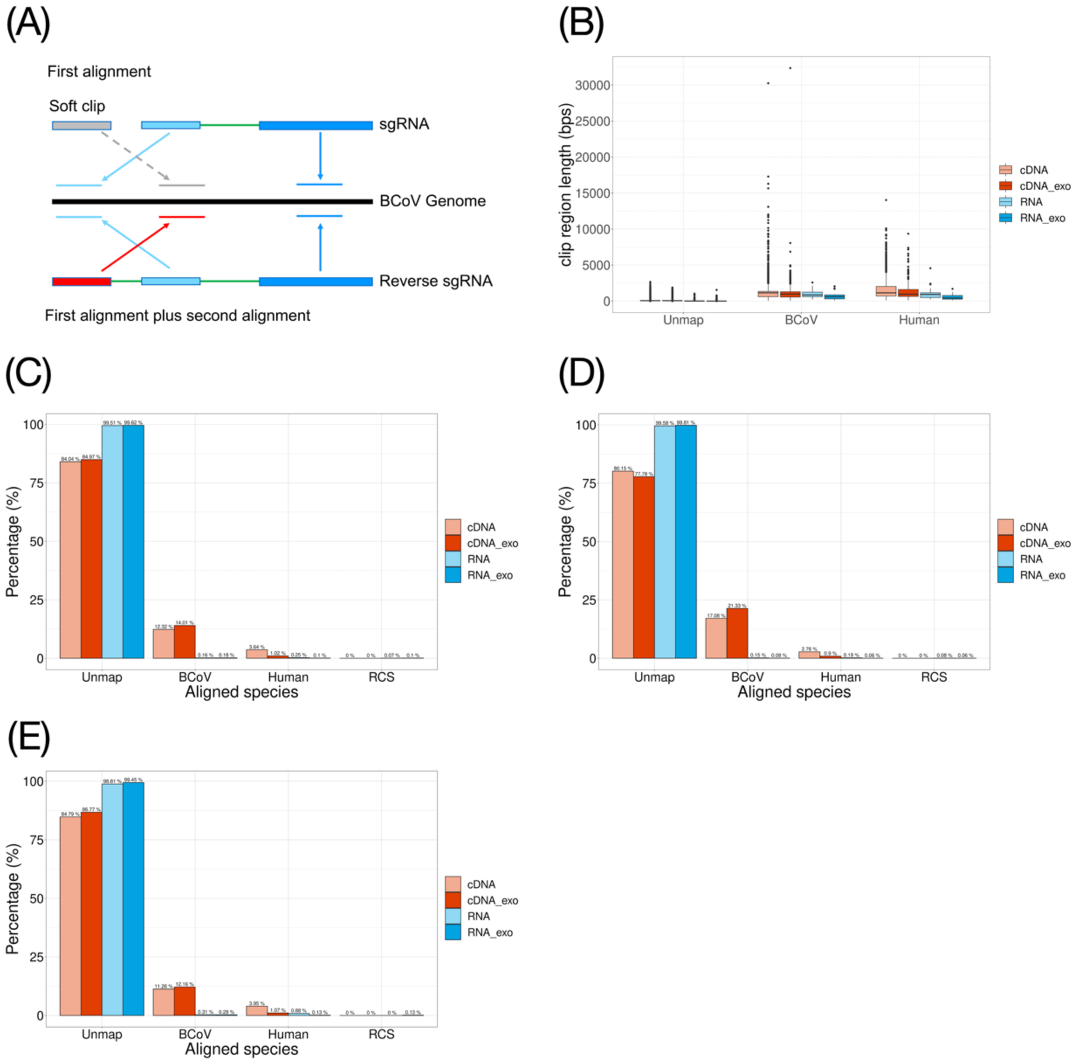

Supplementary Figure S7. (A) Schematic diagram of reverse DVG of bovine coronavirus (BCoV). Since Minimap 2 doesn't allow reversed coordinates when mapping discontinuous RNA transcript with multiple truncations (means that if the second fragment with the coordinate is smaller than the coordinates of the first fragment, the standard procedure of Minimap 2 is to soft clip the second fragment for optimal alignment. The clipped fragment was extracted and re-aligned to BCoV genome. (B) The length distribution of the clipped region among different protocols. (C) The proportion of the clipped reads re-aligned to BCoV genome (U00735.2), human genome (GRCh38), or unmethylated RNA calibration standard (RCS). The reads assigned to neither of the three references will be classified as "unmapped". The DVG (D) or sgmRNA (E) clipped sequence reads were re-aligned to three reference genomes. Other than the mapped BCoV genome sequences, chimeric sequences from human or RCS were also identified.

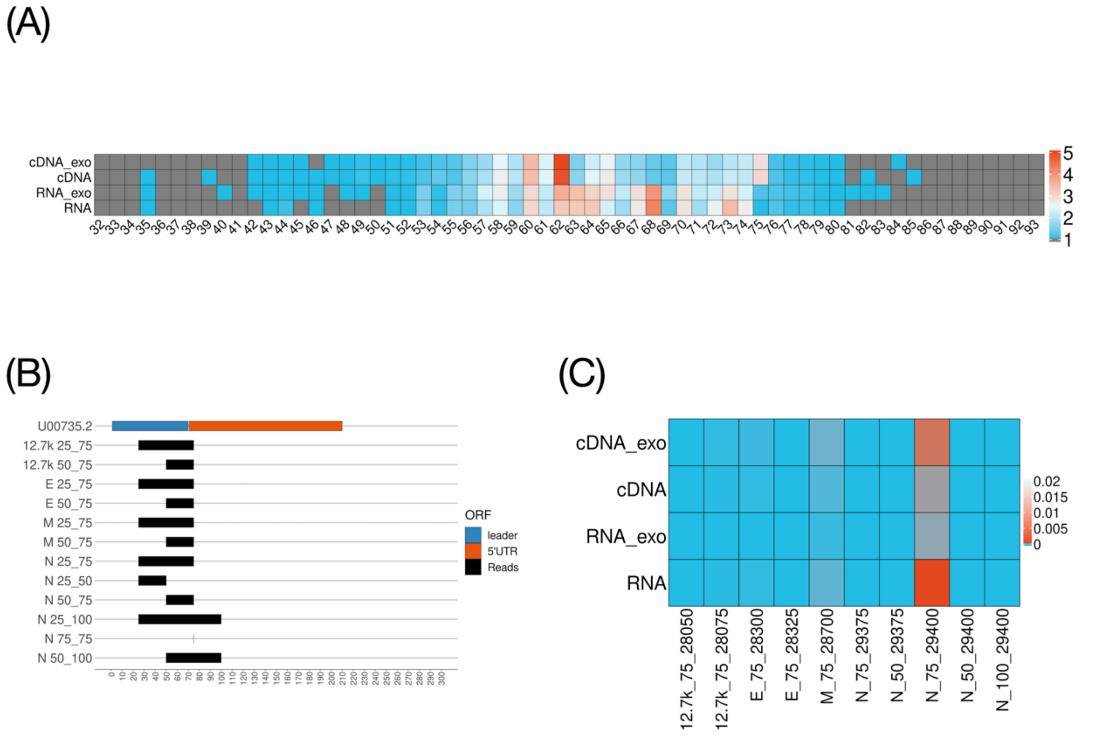

Supplementary Figure S8. (A) The end coordinates of the leader sequences of subgenomic messenger RNA (sgmRNA) from different protocols. The counts of the reads with the same ending coordinates are calculated as log(percentage) and expressed as a heat map. (B) Schematic presentation of the sgmRNA with 5’ terminal containing leader sequence, which fused with 3’ genomic terminal to be translated into structure proteins, including 32k, HE, 12.7k, E, M, N. (C) The end coordinates of the leader sequences were binned in every 25 nucleotides and the expression level based on the counts of reads were shown as a heat map for a different protocol.

Supplementary Table S1. Oligonucleotide primers used in this study

oligonucleotides	length	orientation	Genomic position	sequences (5' to 3')
BCoV 1536 (-)	21	forward	1536-1557	Gagtgcagcaggttatggtgg
BCoV 9938-9960 (-)	23	forward	9938-9960	Cattcttgcaatctggtattgtg
BCoV 11008-11032 (-)	25	forward	11008-11032	Gttataagtttgaccatgttgttgg
BCoV 13343-13366 (-)	24	forward	13343-13366	Ggttcggggtacgagtgtagatgc
BCoV 29461-29483 (+)	23	reverse	29461-29483	Ctgatcggcccacttaaggatgc

Supplementary Table S2. Comparison of total reads, mapping results, reads length, quality score comparing k-mer at 8 and 20 among four different protocols

		K=8					K=20				
		**BCoV**	**Unmap**	**Median aligned length (bp)**	**Median quality score**	**Mapped nucleotide (%)**	**BCoV**	**Unmap**	**Median aligned length**	**Median quality score**	**Mapped nucleotide (%)**
**Protocol #1**	**Total reads**										
RNA_exo1	894,744	257,267 (28.7%)	637,477 (71.25 %)	1,686	10.35	91.24 %	255,486 (28.55 %)	639,258 (71.45 %)	1,640	10.33	91.35 %
RNA_exo2	109,527	24,355 (22.23%)	85,172 (77.76 %)	1,677	10.26	90.74 %	24,179 (22.07 %)	85,348 (77.92 %)	1,638	10.22	90.86 %
subtotal (%)	1,004,271	281,622 (28.04%)	722,649 (71.96 %)				139,832 (25.31 %)	724,606 (72.15 %)			
**Protocol #2**	**Total reads**										
RNA1	812,991	317,746 (39.08%)	495,245 (60.92 %)	1,654	8.35	85.78 %	309,886 (38.12 %)	503,105 (61.88 %)	1,635	8.35	85.88 %
RNA2	178,476	73,999 (41.46%)	104,477 (58.54 %)	1,657	7.85	83.86 %	71,292 (39.03 %)	107,184 (60.06 %)	1636	7.86	84.00 %
subtotal (%)	991,467	391,745 (39.51%)	599,722 (60.49 %)				190,589 (39.03 %)	610,289 (61.55 %)			
**Protocol #3**	**Total reads**										

cDNA_exo1	208,409	42,138 (20.21%)	166,271 (79.78 %)	849	9.59	91.39 %	42,014 (20.16 %)	166,395 (79.84 %)	847	8.48	91.66 %
cDNA_exo2	494,479	119,676 (24.2%)	374,803 (75.80 %)	905	12.99	95.74 %	120,160 (24.30 %)	374,319 (75.70 %)	906	10.68	95.95 %
subtotal (%)	702,888	161,814 (23.02%)	541,074 (76.98 %)				81,087 (22.23 %)	540,714 (76.93 %)			
**Protocol #4**	**Total reads**										
cDNA1	827,842	226,904 (27.4%)	600,938 (72.59 %)	1,476	11.66	94.04 %	228,586 (27.61 %)	599,256 (72.39 %)	1,496	9.96	94.21 %
cDNA2	142,109	45,516 (32.03%)	96,593 (67.97 %)	983	10.30	92.76 %	45,602 (32.09 %)	96,507 (67.91 %)	994	8.85	93.05 %
subtotal (%)	969,951	272,420 (28.09%)	697,531 (71.91 %)				137,094 (28.26 %)	695,763 (71.73 %)			
